# A multi gene sequence-based phylogeny of the Musaceae (banana) family

**DOI:** 10.1186/1471-2148-11-103

**Published:** 2011-04-16

**Authors:** Pavla Christelová, Miroslav Valárik, Eva Hřibová, Edmond De Langhe, Jaroslav Doležel

**Affiliations:** 1 Centre of the Region Haná for Biotechnological and Agricultural Research, Institute of Experimental Botany, Sokolovská 6, 772 00 Olomouc, Czech Republic; 2 Laboratory of Tropical Crop Improvement, Katholieke Universiteit Leuven, Kasteelpark Arenberg 13, Leuven, Belgium

## Abstract

**Background:**

The classification of the Musaceae (banana) family species and their phylogenetic inter-relationships remain controversial, in part due to limited nucleotide information to complement the morphological and physiological characters. In this work the evolutionary relationships within the Musaceae family were studied using 13 species and DNA sequences obtained from a set of 19 unlinked nuclear genes.

**Results:**

The 19 gene sequences represented a sample of ~16 kb of genome sequence (~73% intronic). The sequence data were also used to obtain estimates for the divergence times of the Musaceae genera and *Musa *sections. Nucleotide variation within the sample confirmed the close relationship of *Australimusa *and *Callimusa *sections and showed that *Eumusa *and *Rhodochlamys *sections are not reciprocally monophyletic, which supports the previous claims for the merger between the two latter sections. Divergence time analysis supported the previous dating of the Musaceae crown age to the Cretaceous/Tertiary boundary (~ 69 Mya), and the evolution of *Musa *to ~50 Mya. The first estimates for the divergence times of the four *Musa *sections were also obtained.

**Conclusions:**

The gene sequence-based phylogeny presented here provides a substantial insight into the course of speciation within the Musaceae. An understanding of the main phylogenetic relationships between banana species will help to fine-tune the taxonomy of Musaceae.

## Background

The global annual production of bananas and plantains (*Musa *spp.) amounts to > 120 Mt [1], making this species one of the world's most important fruit crops. As well as their prominence as a dessert fruit, they provide a vital source of carbohydrates to many inhabitants of the humid tropics. *Musa *production, like that of all crop species, is endangered by a range of pests and diseases, affecting both the yield and quality of the fruit. While the large-scale commercial plantations can secure production by frequent applications of fungicide and pesticide, this form of crop management is increasingly recognized as environmentally irresponsible. Meanwhile, smallholders, who together account for at least 85% of world production, can seldom afford the expense of chemical control, and their crop remains vulnerable to diseases and pests. Improvement of cultivated banana via breeding is hampered by the absence of sexual reproduction and narrow genetic basis. As a result, attention has turned to non-cultivated wild relatives as sources of new genes for banana improvement. This, underlines a renewed interest to analyze and conserve genetic diversity within *Musa *spp., which in turn has raised a number of questions related to their taxonomy.

The banana family (Musaceae) has been assigned to the order Zingiberales in the clade commelinids in the monocots [2] and has been conventionally divided into the three genera *Musa, Ensete *and *Musella*. The genus *Musa *is characterized by a set of morphological descriptors, and has a basic chromosome number (*x*) of 9, 10 or 11. The genus has been sub-divided into the four sections *Eumusa *(*x *= 11; comprising most of the cultivated species), *Rhodochlamys *(*x = *11), *Australimusa *(*x *= 10) and *Callimusa *(*x *= 9, 10) [3,4]. More recently, Argent [5] added a fifth section, *Ingentimusa *(*x *= 7), containing just a single species *M. ingens*. However, since this one species (x = 7) grows within the *Australimusa *region (New Guinea), its section-status is not evident when compared to *M. beccarii *(x = 9), which grows in the *Callimusa *region (Borneo) and remains classified as a *Callimusa*.

With the application of DNA-based tools, this conventionally-based taxonomy has become increasingly difficult to justify. Thus, based on RFLP genotyping, Gawel *et al. *[6] proposed a merger between *Eumusa *and *Rhodochlamys*, a suggestion consistent with nuclear genome sizes and the distribution of rDNA loci [7], as well as with the phylogenetic analysis based on the ITS and organellar DNA [8]. Jarret and Gawel [9] further proposed combining *Australimusa *and *Callimusa *into a single section, a suggestion supported by AFLP genotypes acquired by Wong *et al*. [10]. However, the results of AFLP genotyping led Ude et al. [11] to argue that the conventional taxonomy of *Musa *was in fact tenable.

The ease of DNA sequencing has revolutionized phylogenetic methodology. The most frequent targets for this type of analysis have been extra-nuclear DNA i.e. chloroplast and mitochondrial genes [12-16] and the internal transcribed spacers (ITS) separating the tandem organized ribosomal genes in the 45S rDNA locus [17-19]. The prevalently uniparental mode of inheritance of the chloroplast and mitochondrion limits to some extent the usefulness of extra-nuclear sequences, and moreover, it has been established that this DNA tends to evolve more slowly than do the nuclear genes, which presents difficulties in employing it for phylogenetic purposes [20]. Concerted evolution [21], a bias due to analyzing a single locus and hidden paralogy all militate against relying solely on ITS variation for molecular systematics and evolutionary analysis [22,23].

Single and low copy nuclear gene sequences are thought to provide a higher level of discrimination than either extra-nuclear genes or ribosomal spacers [24-26]. The lower frequency of informative sites within these sequences can, however, prevent their use for the resolution of phylogeny both at lower taxonomic levels and among rapidly diversifying lineages. The greater resolving power of low copy nuclear sequence has been recently demonstrated in rice [27]. Low copy nuclear genes also suffer less homoplasy than does ITS [22] and are seldom subjected to concerted evolution. Intronic sequence is particularly useful, since the level of selection pressure on its non-coding DNA is relaxed [28]. The major drawback to the use of low copy sequence is the need to distinguish between paralogs and orthologs. As yet in the Musaceae family, however, all published sequence-based phylogenetic studies have targeted extra-nuclear and/or ribosomal DNA sequence.

The phylogeny of the Musaceae remains controversial. Typing via organellar and ribosomal DNA has been employed by Boonruangrod *et al*. [29,30]. Li *et al*. [8] and Liu *et al*. [31] applied sequence analysis of ribosomal ITS coupled with the chloroplast gene evidence. More generally, evolutionary relationships within the monocotyledonous species [32-34] and in the Zingiberales in particular [35,36], have produced date estimates for the divergence of the Musaceae (61-110 Mya) and the genus *Musa *(51 Mya). Based on a study of genome duplication, Paterson *et al*. [37] suggested that the divergence of *Musa *occurred 142 Mya, although this estimate was conceded to require further sequence information before it could be accepted. Clearly, a more robust picture of banana phylogeny and divergence time requires a systematic sampling of gene sequences distributed throughout the genome. Thus, we set out to clarify main frame of evolutionary relationships within the Musaceae, and to date the divergence of particular *Musa *sections, using a set of single or low copy nuclear gene sequences.

## Methods

### Taxon sampling

The sample of Musaceae species included representatives of *Musella, Ensete *and each of the four *Musa *sections (Table 1). *Strelitzia nicolai *Regel et Koern (family Strelitziaceae, order Zingiberales) was chosen to serve as an outgroup due to its relatively close relationship to the Musaceae family and the highest efficiency of amplification of selected gene markers. Sampling of additional outgroup species was abandoned after a series of preliminary tests, which revealed major difficulties with the amplification of selected genes (data not shown). *In vitro *rooted *M. balbisiana *'PKW' plants were donated by François Côte (CIRAD, Guadeloupe, French West Indies) and *Musella lasiocarpa *plants were purchased from a commercial nursery. The other entries were obtained from International Transit Centre (ITC, Catholic University, Leuven, Belgium) in the form of *in vitro *rooted plants. All plant materials were maintained in a greenhouse after their transfer to soil. Leaf tissue of *S. nicolai *was provided by Dr. M. Dančák (Palacký University, Olomouc, Czech Republic). Genomic DNA was extracted from young leaf tissues using Invisorb^® ^Spin Plant Mini kit (Invitek, Berlin, Germany), following the manufacturer's instructions.

**Table 1:** A priori taxonomic status of the panel of 13 Musaceae entries

International Transit Centre (ITC) code	Accession name	Genus	Section	Species/Group [Authority]	Subspecies/Subgroup [Authority]	Basic chromosome number (*x*)
0249	Calcutta4	*Musa*	*Eumusa*	acuminata [Colla] *	burmannica [N.W.Simmonds]	11
0728	Maia Oa	*Musa*	*Eumusa*	acuminata [Colla] *	zebrina [Van Houtte ex. Planch.]	11
1120	Tani	*Musa*	*Eumusa*	balbisiana [Colla] #		11
n/a	Pisang Klutuk Wulung	*Musa*	*Eumusa*	balbisiana [Colla] #		11
0637	*Musa ornata*	*Musa*	*Rhodochlamys*	ornata [Roxb.]	ornata	11
1411	*Musa mannii*	*Musa*	*Rhodochlamys*	sanguinea [Hook.f.]		11
0539	*Musa textilis*	*Musa*	*Australimusa*	textilis [Née]	textilis	10
0614	*Musa maclayi *Hung Si	*Musa*	*Australimusa*	maclayi [F.Muell]	maclayi	10
1021	Menei	*Musa*	*Australimusa*	Fe'i	domesticated	10
1070	*Musa beccarii*	*Musa*	*Callimusa*	beccarii [N.W.Simmonds]	beccarii	9
0287	*Musa coccinea*	*Musa*	*Callimusa*	coccinea [Andrews]		10
1387	*Ensete ventricosum*	*Ensete*		ventricosum [Welw.]	ventricosum	9
n/a	*Musella lasiocarpa*	*Musella*		lasiocarpa [Franch.]		9

* M. acuminata species, hereafter also reffered to as the A - genome group

# *M. balbisiana *species, hereafter also reffered to as the B - genome group

### Target gene selection and primer design

The gene sequences targeted for phylogenetic analysis were selected from the collection of banana ESTs deposited in GenBank as of March 30, 2009. The threefold basis for the choice of genes was that they were single copy, that their genomic locations spanned the entire genome and that they contained at least one intron. Genes belonging to the same gene family were avoided. These criteria were applied by reference to their rice orthologs, which were identified by BLAST analysis [38], using a threshold of e^-10^. To maximize dispersion across the banana genome, we chose genes whose rice orthologs mapped to different chromosome arms. Gene structure in banana was assumed to be identical to that in rice. Primers (see Additional File 1) were designed to amplify intron-spanning gene fragments in the panel of Musaceae species and *S. nicolai*, following Lessa [39]. Primers which either failed to amplify or amplified multiple fragments from any one of the 13 Musaceae entries were discarded. The final set comprised 19 genes (Table 2), sampling each of the rice chromosome arms except the long arms of chromosomes 4, 5, 11 and 12, and the short arm of chromosome 12. Nine of the 19 primer pairs (Table 2) amplified successfully from *S. nicolai *template.

**Table 2:** Identity and sequence details of the set of 19 genes targeted for phylogenetic purposes

Candidate gene designation	**Original *Musa *EST (NCBI accession number) **^**a**^	Blast homology	Corresponding *O. sativa *chromosome	**Homologous region on the *O. sativa *chromosome (bp) **^**b**^	Amplified successfully from the outgroup species (*S. nicolai*)	Intron fraction in the final alignment (%)	**Aligned sequence length (bp) **^**c**^	**Mean GC content (%) **^**c**^
g-1	FF561021.1	ATP:citrate lyase	Chr. 1	11000940 - 11002303	yes	81.5	840 [761]	34.8 [34.6]
g-2	ES433688.1	Stomatal cytokinesis defective protein	Chr. 1	22165522 - 22168085	yes	39.0	776 [720]	39.4 [39.5]
g-3	FF558855.1	Electron transport protein SCO1/SenC family protein	Chr. 2	3229271 - 3231294	yes	90.8	882 [869]	33.3 [33.4]
g-4	ES437588.1	Putative non-phototropic hypocotyl 3 (NPH3)	Chr. 2	21628715 - 21631333	no	33.5	529	62.7
g-5	ES436517.1	Endoribonuclease dicer homolog	Chr. 3	1177218 - 1178862	yes	45.3	890 [827]	36.9 [37.5]
g-6	FF559301.1	CASP protein-like	Chr. 3	28468384 - 28470215	yes	75.6	897 [863]	37.6 [37.8]
g-7	FF559765.1	Zeaxanthin epoxidase	Chr. 4	22349032 - 22350073	no	70.5	909	40.4
g-8	ES437560.1	Na/H antiporter	Chr. 5	2717353 - 2718249	no	33.2	487	47.6
g-9	FF561211.1	Protein of unknown function; DUF89 family protein	Chr. 6	12551039 - 12553284	yes	86.9	1045 [942]	33.5 [33.5]
g-10	ES436526.1	T-complex protein 1, eta subunit (TCP-1-eta)	Chr. 6	28301038 - 28302942	no	69.3	1154	34.1
g-11	ES434922.1	NAD+ synthase domain containing protein	Chr. 7	3637535 - 3641458	yes	54.5	794 [747]	36.5 [36.3]
g-12	FF561580.1	Ribosomal protein s6 RPS6-2	Chr. 7	25669518 - 25671532	no	67.7	755	39.7
g-13	FF559780.1	mRNA capping enzyme, large subunit family protein	Chr. 8	4670797 - 4673706	no	91.2	1433	37.2
g-14	FF560522.1	Methylcrotonyl-CoA carboxylase beta chain	Chr. 8	20354213 - 20356265	no	87.8	767	34.1
g-15	FF560378.1	Annexin-like protein	Chr. 9	13652033 - 13655072	no	92.5	737	38.7
g-16	ES436518.1	Succinoaminoimidazole-carboximide ribonucleotide synthetase family protein	Chr. 9	17688901 - 17690929	no	72.0	587	36.2
g-17	FF558349.1	Methionine aminopeptidase 1	Chr. 10	18996256 - 18997164	no	73.4	746	35.9
g-18	FF559189.1	Initiation factor 2B family protein	Chr. 10	12576657 - 12577797	yes	95.9	915 [915]	34.1 [34.1]
g-19	ES436684.1	DNA polymerase delta catalytic subunit	Chr. 11	4368226 - 4369480	yes	74.0	878 [760]	35.4 [36.2]

^a ^The *Musa *EST sequence from which the corresponding gene fragment was derived.

^b ^Genomic location in rice.

^c ^Data in square brackets include the outgroup *S. nicolai*.

### Gene fragment amplification, cloning and seqeuncing

A standard amplification protocol was applied to each of the 19 primer pairs. Each reaction contained 40 ng template, with the PCR program composed of an initial denaturation step (94°C/5 min), followed by 35 cycles of 94°C/30 s, 57°C/30 s and 72°C/35 s, and ending with an extension step of 72°C/10 min. Amplicons were treated with exonuclease/alkaline phosphatase (ExoSAP-IT^®^, USB, Cleveland, OH, USA) and then either sequenced directly, or first cloned into the TOPO vector (Invitrogen, Carlsbad, USA) before sequencing. Cycle sequencing was performed on three independent amplicons per gene target, using a BigDye^® ^Terminator v3.1 Cycle Sequencing kit (Applied Biosystems, Foster City, USA), following the manufacturer's instructions. Sequencing reaction products were purified using a CleanSEQ kit (Agencourt Bioscience Corp., Beckman Coulter, Beverly, USA), and then separated on an ABI 3730Xl DNA analyzer (Applied Biosystems). All the resulting sequences have been deposited within GenBank [GenBank: HM118565-HM118820]. Raw sequence data were assembled and edited using DNA Baser v2 software [40]. Consensus sequences were aligned by ClustalW [41] using default parameters, as implemented in the MEGA4 software package [42]. Multiple DNA sequence alignments were inspected and any ambiguously aligned segments were removed prior to phylogenetic analysis.

### Phylogenetic reconstruction

Maximum likelihood (ML), maximum parsimony (MP) and Bayesian inference (BI) methods were applied to infer phylogenetic relationships. Sequence gaps were treated as missing data. Two datasets were considered - the first (dataset A) consisted of all 19 gene fragments across the 13 Musaceae entries, but not *S. nicolai*, and the second (dataset B) comprised nine gene fragments across all the entries. Exonic and intronic sequences were analyzed separately in a similar fashion. MP and ML analyses were performed using PAUP* v4.0b software [43]. The most parsimonious tree for each dataset was found by a heuristic search of 1,000 random sequence-addition replicates by means of a tree-bisection-reconnection (TBR) branch swapping algorithm. The strict consensus tree was rooted by *S. nicolai *as an outgroup or, where no sequence was obtainable from this species, by *E. ventricosum*. Statistical support for individual nodes was estimated from 1,000 bootstrap replicates. The best model, as suggested by MrModeltest v2.3 software [44], based on the Akaike information criterion (AIC, see Table 3) was implemented in the ML and BI parameter settings for each target gene fragment, as well as for the full datasets. The ML-based optimal tree was derived from 100 simple sequence-addition replicates using TBR branch swapping, and bootstrap support values were calculated from 100 replicates. BI analysis was conducted in BEAST v1.4.8 [45] using four independent Markov Chain Monte Carlo (MCMC) runs, starting from a randomly chosen topology, and run for 1,000,000 generations, with sampling every 1,000 generations. Logfile outputs were inspected in Tracer [45] software to confirm convergence. Treefiles from individual runs were combined by LogCombiner [45] software. The maximum clade credibility tree and corresponding posterior probabilities were calculated using TreeAnnotator [45] software, after removal of the 25% burn-in samples. The phylogenetic trees generated were graphically adjusted in FigTree v1.3.1 software [46].

**Table 3:** Evolutionary models as selected by MrModeltest v2.3 software for each of the individual gene fragments and for the combined datasets using AIC criteria

Gene/gene data set	AIC best model fit	Gamma distribution shape parameter	Ti/Tv ratio
g-1	HKY+I	Equal rates	2.3975
g-1 *	HKY+I	Equal rates	2.0159
g-2	HKY	Equal rates	1.4707
g-2 *	HKY+I	Equal rates	1.4748
g-3	HKY	Equal rates	1.6034
g-3 *	HKY	Equal rates	1.5926
g-4	GTR	Equal rates	n/a
g-5	HKY+I	Equal rates	0.9858
g-5 *	HKY+G	0.7707	1.3060
g-6	HKY	Equal rates	1.6034
g-6 *	HKY+I	Equal rates	1.9435
g-7	HKY	Equal rates	2.0482
g-8	K80+G	0.4266	1.2698
g-9	HKY+G	0.3713	1.2549
g-9 *	HKY+G	1.4564	1.1334
g-10	HKY+G	0.5641	1.7196
g-11	HKY+G	0.2939	1.1779
g-11 *	HKY+G	0.7744	1.2061
g-12	HKY+I	Equal rates	1.8313
g-13	HKY+I	Equal rates	1.5462
g-14	HKY	Equal rates	1.1717
g-15	HKY+I	Equal rates	1.1507
g-16	HKY+I	Equal rates	1.1031
g-17	HKY+G	1.0609	1.7507
g-18	HKY	Equal rates	1.4334
g-18 *	HKY	Equal rates	1.4658
g-19	HKY+I	Equal rates	1.4589
g-19 *	HKY+I	Equal rates	1.1127
data set A	HKY+G	0.9572	1.4147
data set B	HKY+G	0.6449	1.4868
exons	HKY+G	0.1595	2.0983
introns	GTR+G	1.0173	n/a

* including the outgroup *S. nicolai*

### Systematic bias and congruence testing

The incongruence length difference (ILD) test [47] (implemented in PAUP* v4.0b as the partition homogeneity test) was applied to estimate the level of potential incongruence in the data. The data set was partitioned into individual genes and analyzed under heuristic search with 1000 replicates. A χ^2 ^test for base composition homogeneity across taxa was conducted in TREE-PUZZLE v5.2 [48] software. The level of nucleotide substitution saturation was evaluated in DAMBE [49] software by plotting transitions and transversions against pairwise genetic distance. ML mapping using the quartet puzzling method [50] was applied to investigate whether the phylogenetic information content of the data was sufficient for inference purposes. ML mapping was also performed within TREE-PUZZLE v5.2 software with all possible quartets, applying the corresponding evolutionary model and exact model parameter estimation settings.

### Dating of nodes

BEAST software v1.4.8 software was used to estimate the divergence times for the major Musaceae clades. This approach has the advantage of simultaneous estimation of substitution model parameters, topology, branch lengths and fossil-based date calibration, using the Bayesian inference and MCMC method. Calibration was based on the carbon dating of *Ensete oregonense *fossil seeds, given as 43 Mya according to Manchester and Kress [51]. The analysis was conducted over four independent MCMC runs, each consisting of 1,000,000 generations under the relaxed clock model, with an uncorrelated lognormal distribution. The fossil calibration was set as the most recent common ancestor (t_MRCA_) parametric tree prior. The results were retrieved after combining the individual MCMC runs' tree files and the maximum clade credibility tree was constructed after the initial 25% burn-in generations were discarded.

## Results and Discussion

### Taxon and gene sampling

The amount of available sequence information for *Musa *species is confined at present and hence the development of low-copy gene markers for phylogenetic studies in this species has been laborious and time consuming. Despite this, we were able to develop 19 markers from gene regions. Only single- or low copy genes were selected with expected random distribution in the genome of *Musa *to make sure that unlinked loci are compared. As the genome sequence of *Musa *is not yet available, the selection of random distributed loci assumed colinearity with the rice genome [52,53].

The 19 gene-based markers [GenBank: HM118565-HM118820] developed and used in the present study represent until now by far the largest set of gene markers ever used in the *Musaceae*. Ideally, a phylogenetic study should comprise all taxa and a high number of unlinked DNA markers. However, from practical reasons these numbers are reduced and, in fact, may not be necessary. While some authors argue that incomplete taxon sampling has a negative impact on the phylogenetic accuracy [54,55], other authors do not support this view and prefer increasing the number of nucleotide characters sampled over the number of taxa in order to reveal the correct phylogeny without a major distortion of accuracy of the main evolution relationships [56-58]. Here, we favored the latter approach with partial taxon sampling of representatives [stratified sampling; [59]], rather than analyzing a few genomic loci on a large set of species. However, if felt necessary, the marker set developed in this work can be easily applied in other species and subspecies of Musaceae.

### Sequence data characterization and systematic bias testing

The 19 gene fragments covered a length of 16,012 bp, of which 26.9% was exonic. The genic sequences were treated independently as a single-gene data and in two matrixed-modes according to the ability to amplify the genes from the outgroup species *S. nicolai *(see Table 2 for details); namely the dataset A (containing all 19 gene sequences from 13 genotypes, excl. *S. nicolai*) and the dataset B (containing sequences of 9 genes from all 14 genotypes, incl. the outgroup species *S. nicolai*). Dataset A (all 19 fragments from the 13 Musaceae entries) was based on 16,012 bp of sequence, of which 1,056 bases were informative, while dataset B (nine gene fragments from the Musaceae entries plus *S. nicolai*) was based on 7,404 bp of sequence, which included 492 informative sites. The χ^2 ^test used to detect heterogeneity in base composition indicates that there was no significant variation in the AT/GC content among species for individual genes (P = 0.382-1.000). The overall reduced proportion of GC in most of the sequences (see Table 2) may be an artifact of the deliberate maximization of intronic sequence in the sample, since plant intronic sequence has an AT bias [60]. The GC content of the intronic fraction was 34.6%, compared to 45.0% in the exonic fraction.

**Figure 1:**
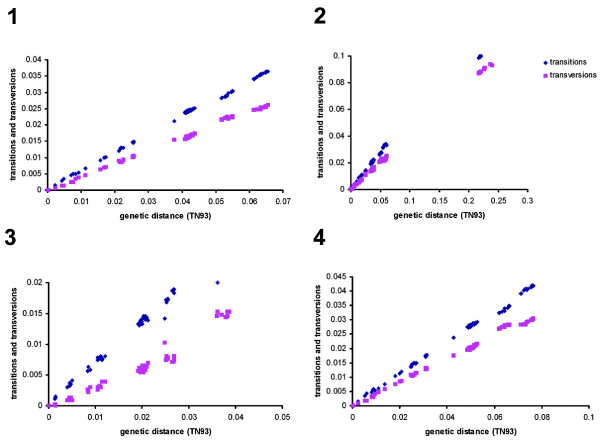
**Transitions and transversions versus divergence plots**. The estimated number of transitions and transversions for each pairwise comparison was plotted against the genetic distance calculated with the TN93 model [80] for (1) dataset A, (2) dataset B, (3) exonic sequence, (4) intronic sequence. Transitions outnumber transversions along with the linear increase in the genetic distance. This pattern can be interpreted as indicating that substitution saturation has not been reached, so that the data can be expected to provide sufficient phylogenetic signal.

Nucleotide sequences are considered to be phylogenetically informative until they reach the substitution saturation. At this point, it is no longer possible to deduce whether an observed similarity between a pair of sequences results from their common ancestry or whether this has occurred by chance [61]. To avoid the inclusion of non-informative sequence, the level of substitution saturation was evaluated by plotting transitions and transversions against the genetic distance for both datasets A and B, as well as for the exonic and intronic sequence separately. This procedure showed that the frequency of both transitions and transversions increased linearly along with divergence (Figure 1) with transitions outnumbering transversions. This indicates that the saturation plateau was not reached, and the data still retained sufficient phylogenetic signal.

The constancy of the evolutionary rate was verified using a relative rate test, which revealed some heterogeneity in the sequences (data not shown). However, after a re-analysis based on RY-coded (purines/pyrimidines) sequence, which ignores transitions by focusing on the slower evolving transversions [62], the topologies generated were similar to those obtained from the full nucleotide sequence data. This implied that the rate heterogeneity was not large enough to significantly bias the deduced phylogenies.

### Phylogenetic reconstruction based on individual gene fragments

**Figure 2:**
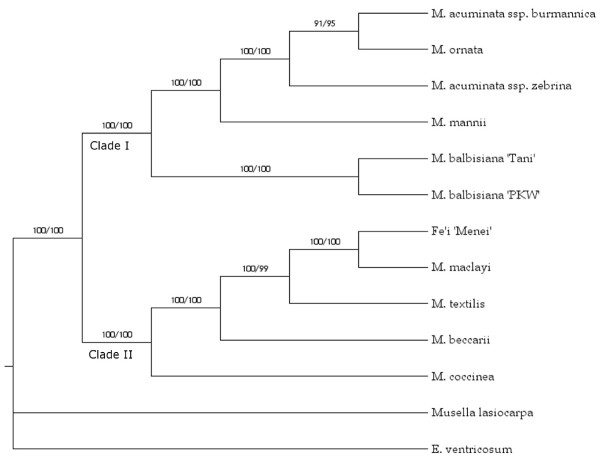
**A phylogenetic tree based on dataset A sequence, with *E. ventricosum *as outgroup**. Values above the branches indicate bootstrap support for MP and ML, respectively. Clades I and II indicate known taxonomic divisions within the Musaceae. Clade I: *Eumusa *(*M. acuminata *[A genome] and *M. balbisiana *[B genome]) plus *Rhodochlamys *(*M. mannii *and *M. ornata*) entries. Clade II: *Australimusa *(*M. textilis, M. maclayi *and Fe'i) and *Callimusa *(*M. coccinea *and *M. beccarii*) entries.

The reconstruction of phylogenetic relationships between the selected taxa representing the Musaceae family was performed by two different criterion-based methods (maximum parsimony; MP and maximum likelihood; ML) and by a third complementary approach based on the Bayesian inference method (BI). Data were first executed in MrModeltest v.2.3 [44] in order to select the most appropriate model of evolution to be used for phylogenetic analyses. The Akaike Information Criterion was chosen [63] to be implemented in maximum likelihood and Bayesian analysis, as it was reported to have preferable performance in model selection compared to likelihood ratio tests [64]. The evolutionary models selected for the phylogenetic reconstruction are detailed in Table 3. The MP analysis based on the individual gene fragment sequences produced more than one most parsimonious tree for eight of the 19 sequences (Additional File 2). In 15 of the 19 phylogenies there were unresolved polytomies. Clades I (*Eumusa *+ *Rhodochlamys*) and II (*Australimusa *+ *Callimusa*) were fully recovered (Figure 2), except for gene fragment g-4, the sequence of which comprised one of the shortest intron sequences and the lowest proportion of phylogenetically informative positions. A similar result was obtained by ML analysis, in which partially resolved phylogenies applied to 15 of the 19 sequences, with an altered topology appearing within either clade I or II for gene fragments g-5, g-12, g-17 and g-19 (Additional File 2).

The BI analysis generated fully resolved phylogenies, albeit with topology alterations within clades I and II. The level of internal resolution within clades I and II varied according to the phylogenetic informativeness of the sequences. Unresolved relationships emerged within both clades I (between *M. acuminata, M. mannii *and *M. ornata*), and II (between *M. textilis*/*M. maclayi*/Fe'i and *M. beccarii*/*M. coccinea*). When the phylogenetic content of the sequences was evaluated by the likelihood-mapping approach, it was clear that each of the single gene fragment-based phylogenies contained a significant fraction of unresolved quartets (Table 4), showing that a single sequence is insufficient for making inference regarding evolutionary relationships. However, for both of the combined datasets A and B, there were no unresolved or partially resolved quartets and thus we investigated a possibility of combining individual gene data into a single data set for the phylogenetic reconstruction.

**Table 4:** Results of the likelihood-mapping based on the quartet puzzling algorithm

Gene/gene data set	Partially resolved quartets (%)	Unresolved quartets (%)
g-1	0.0	28.3
g-1 *	0.5	12.3
g-2	0.0	4.1
g-2 *	1.7	11.7
g-3	1.7	5.2
g-3 *	1.3	3.7
g-4	5.6	9.0
g-5	2.7	9.9
g-5 *	1.9	7.8
g-6	0.0	6.3
g-6 *	2.8	9.9
g-7	0.2	1.7
g-8	1.0	9.0
g-9	3.5	9.1
g-9 *	1.9	11.2
g-10	0.2	5.7
g-11	5.5	6.9
g-11 *	5.0	8.2
g-12	0.7	1.4
g-13	0.4	1.4
g-14	4.2	3.6
g-15	1.4	2.7
g-16	5.5	14.8
g-17	0.3	6.4
g-18	0.5	1.1
g-18 *	0.4	1.1
g-19	0.0	2.2
g-19 *	1.8	11.7
data set A	0.0	0.0
data set B	0.0	0.0
exons	0.4	0.8
introns	0.0	0.0

* including the outgroup *S. nicolai*

The proportion of unresolved and partially resolved quartets is shown for phylogenies based both on single gene fragments, and on the combined data.

Based on the ILD analysis, the individual gene fragment partitions were highly incongruent (P < 0.001) and thus not directly combinable. However, it has been suggested that the ILD test should not be used as an exclusive measure of data partition combinability [65], as it is known to be susceptible to both types I [false positives; [66]] and II [false negatives; [67]] error. When Rokas *et al*. [68] combined sequence data derived from a set of different genes, conflicting signals from individual gene sequences were resolved and the resulting phylogeny was strongly supported. The joint use of a set of gene sequences for phylogenetic inference depends largely on nucleotide composition bias and substitution saturation [61]. Since the χ^2 ^test applied to the Musaceae sequence data indicated the absence of any base composition bias, and substitution saturation of the aligned sequences could be excluded (Figure 1), the combined set of gene fragment sequences was then used for phylogenetic reconstruction.

### Phylogenetic reconstruction based on the combined sequence data

MP analysis of dataset A yielded a single fully resolved most parsimonious tree (length = 2333; CI = 0.8678 excluding non-informative characters; RI = 0.9337; RC = 0.8648) with significantly high level of bootstrap support for each of the individual branches (Figure 2). The internal branches among the *M. acuminata *accessions and the *Rhodochlamys *species, as well as within the *Australimusa*/*Callimusa *clade were dichotomous. The ML analysis supported an identical tree topology with high bootstrap support values. Although the BI analysis also produced a fully resolved tree with a high posterior probability for all nodes (Additional File 3), the monophyly of *Ensete *and *Musella *at the genus level was not supported. Due to the lack of an outgroup for dataset A, *E. ventricosum *was used as a surrogate, a choice which probably accounted for the MP and ML-based phylogenies. The fact that these phylogenies were likely artefactual was confirmed by the use of the midpoint rooting method, which generated the same topology as emerged from the BI analysis and from dataset B (see below).

**Figure 3:**
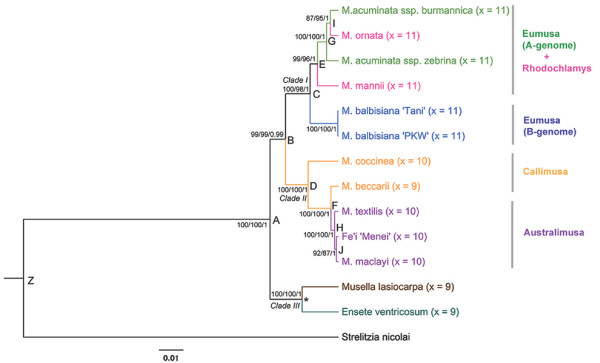
**A phylogenetic tree based on dataset B sequence, with *S. nicolai *as outgroup**. Values above and below the branches indicate bootstrap support for MP and ML, and the posterior BI probability, respectively. Clade I: *Eumusa *(*M. acuminata *[A genome] and *M. balbisiana *[B genome]) plus *Rhodochlamys *(*M. mannii *and *M. ornata*) entries. Clade II: *Australimusa *(*M. textilis, M. maclayi *and Fe'i) plus *Callimusa *(*M. coccinea *and *M. beccarii*). entries. Clade III: *Musella *+ *Ensete *genus. The lettering (A-J) attached to the secondary nodes refers to divergence times, as specified in Table 5.

The MP analysis of dataset B also produced a single most parsimonious tree (length = 2253; CI = 0.7536 excluding non-informative characters; RI = 0.8483; RC = 0.7779) with high bootstrap support for all nodes. The same topology was supported by both the ML and BI analyses (Figure 3), and was the same as emerged from the BI analysis of dataset A (Additional File 3). A similar phylogeny was suggested when the individual gene fragments were analyzed separately with the *S. nicolai *sequence as the outgroup (Additional File 2). Thus the choice of outgroup was clearly responsible for the conflicting phylogenies. Various Zingiberales (Strelitziaceae, Heliconiaceae, Zingiberaceae) species have been selected as outgroups in other taxonomic studies of the Musaceae [8,31,69,70], and some of these have questioned the position of *Musella *as a separate genus. Nevertheless, the evolutionary relationships within *Musa *(clades I + II, Figure 2 and 3) were not affected in either dataset by the choice of either outgroup or rooting method.

In order to assess how much phylogenetic information was contributed by the coding and non-coding fractions, the exonic and intronic sequences were analyzed separately. This was possible given that substitution saturation was not reached in either partition (Figure 1). As expected, the intronic sequence outnumbered the exonic, both in terms of the frequency of variable bases (15.2% *vs *7.1%) and of parsimony informativeness (7.9% *vs *3.3%). The phylogenies reconstructed by ML, MP and BI analysis consisted of a single tree with strong statistical branch support. The trees' topology was identical to that of combined dataset. Thus, the inclusion of non-coding sequence did not introduce erroneous phylogenetic signals, but rather enhanced the robustness of the phylogenetic reconstruction.

### Taxonomic implications of the sequence-based phylogeny

The final topology (Figure 3) confirmed the Musaceae family in general, and the *Musa *genus in particular, to be monophyletic. The monotypic genus *Musella *appeared as a sister species to the *E. ventricosum*. The validity of *Musella *as a genus has been questioned in previous studies and a merger between *Musella *and *Ensete *species has been suggested [31]. On the contrary, the recent study of Li et al. [8] based on ITS and chloroplast loci did not come to a similar definite conclusion and underlined a need for sampling more molecular markers in order to provide the answer. Although more representatives of both of the genera would be necessary to elucidate this issue, the large set of phylogenetic markers presented here provides an excellent tool for addressing this question in future studies.

For many years, *Musa *has been divided into four sections, on the basis of morphological descriptors and basic chromosome number [3]. However, it is important to quote Cheesman's flexible view: "The groups have deliberately been called sections rather than subgenera in an attempt to avoid the implication that they are of equal rank. I am inclined to regard the division between *Eumusa *and *Rhodochlamys *as unessential, though it is convenient to maintain as long as it remains as well marked in the field as it is at present. On the other hand the seed of *Callimusa *almost justifies its segregation as a distinct genus, and would do so were not *Australimusa *intermediate in some characters between it and Eumusa" [3]. Recently, several DNA sequence-based analyses have indeed questioned the validity of some of the four sections. In particular, *Eumusa *and *Rhodochlamys *representatives have been in some cases demonstrated to be more closely related to one another than to their sectional relatives, as was shown for some *Australimusa *and *Callimusa *species [6,7,9,10].

The present data indicate a close relationship between the species of *Rhodochlamys *and *M. acuminata *(*Eumusa*). The position of *M. ornata *within the A-genome group of *Eumusa *section (Figure 3) agrees with the findings of other authors [7,10,31,70], and indicates that *Rhodochlamys *and *Eumusa *are not reciprocally monophyletic. Various *Eumusa *× *Rhodochlamys *hybrids have been observed, and are likely to be numerous in the monsoon region of SE Asia [71]. Although the current molecular data in relation to the morphological observation indicate that the claims for merging of *Rhodochlamys *and *Eumusa *[6,8,10] were justified, final resolution of this issue will require a better representation of species within both sections. The new set of phylogenetic markers developed in this study can be applied easily in future to analyze in detail phylogenetic relationships between and within *Musaceae *taxa.

In contrast to the clustering of *M. balbisiana *with *M. textilis *(section *Australimusa*), as reported by Liu *et al. *[31], the present data identified a clearly separated group of *M. balbisiana *entries within clade I, suggesting that this species is phylogenetically quite distinct from other *Eumusa *species. The distance between *M. acuminata *and *M. balbisiana *appears to be greater than between it and the *Rhodochlamys *species (Figure 3), as has also been noted by others [8,11,31]; these relationships are consistent with conclusions based on cytogenetic and hybridization studies [72,73]. The clear separation between *M. balbisiana *and *M. acuminata *is particularly interesting given that almost all varieties of edible (polyploid) banana are thought to have evolved from natural hybrids between these two species [4].

Based on the gene fragment sequences, *M. textilis *fell, as expected, into the *Australimusa *section within Clade II (Figure 3), which also includes the *Callimusa *species. The two representatives of the section *Callimusa *included in this study differ in the basic chromosome number (Table 1), reflecting the noted controversy of *Callimusa *as a natural section [9,10,74]. *M. beccarii *and *M. coccinea *did not form a strictly separated *Callimusa *cluster; instead, their close relationship to *Australimusa *species was apparent (Figure 3). The only representative of Fe'i bananas (parthenocarpic edible types distributed throughout Pacific islands) in this study appears to be most closely related to *M. maclayi*, in line with Simmonds [71], who considered *M. maclayi *to be a wild progenitor of the Fe'i banana.

### Estimation of time of divergence

The reconstructed phylogeny emerging from dataset A was used to estimate the times of divergence of the major Musaceae clades (Table 5). When the dating was solely constrained by the minimum age of the *Ensete *fossil record, the crown node age of the Musaceae family could be placed in the early Paleocene (69.1 Mya), consistent with the rapid radiation of the Zingiberales in the early Tertiary [32]. A better supported estimate of this time required the inclusion of a relevant outgroup-calibration point within the dataset B, but the lack of fossil records forced us to use of an external calibration point. Estimates for the age of the Zingiberales vary between 88 and 124 Mya [32-34,36]. Here we have adopted the most distant of these dates for the age of the most recent common ancestor of the Musaceae and Strelitziaceae. When this two-point calibration (*Ensete *fossil record and the external calibration with the Zingiberales age) was applied to dataset B, which included the outgroup species, the divergence time of the Musaceae was placed at 61.5 Mya. Estimates of divergence date from both, dataset A and B lie within the Musaceae crown-stem age interval of 61-87 Mya made by Janssen and Bremer [33]. Thus, the estimates (Table 5) emerging from the dataset A comprising nearly doubled amount of phylogenetic information, were considered strongly supported. Based on this data, the rapid diversification of the Zingiberales probably occurred at the Cretaceous/Tertiary boundary (> 65 Mya).

**Table 5:** Estimates of divergence time for species within the Musaceae family

Node description	Node designation (corresponds to Figure 3)	Divergence date (mya)	**HPD range **^**a**^	Reference
Ancestral split	Z	124.0	n/a	[36]
Calibration point (*Ensete oregonense *fossil record)	*	43.0	n/a	[51]
Musaceae	A	69.1	57.8-80.5	present study
*Musa*	B	50.7	40.4-61.5	present study
*M. coccinea *-remaining	D	28.7	21.2-36.6	present study
*Eumusa *B genome-remaining	C	27.9	21.5-34.4	present study
*M. manni *(*Rhodochlamys*)-remaining	E	20.0	15.0-25.9	present study
*Eumusa *A genome	G	11.4	8.6-14.7	present study
*M. beccarii *(*Callimusa*)-remaining	F	8.8	6.1-11.4	present study
*M. ornata*	I	8.8	6.2-11.8	present study
*Australimusa *section-remaining	H	5.2	3.5-6.7	present study
Fe'i - *M. maclayi*	J	2.5	1.4-3.6	present study

^a ^HPD range - the confidence interval of the BEAST analysis expressed as the highest posterior density (HPD) interval.

Despite the fact that the estimated age of the Musaceae family (69 Mya) is much younger than the 110 Mya postulated by Kress and Specht [36], the two estimates for the age of the *Musa *genus (50.7 Mya and 51.4 Mya) are indistinguishable. As the Musaceae are over-represented in our sample (as compared to other Zingiberales families), the current estimate probably represents a minimal age for the radiation of the Musaceae. The present data can be used to date the speciation events within both *Australimusa*/*Callimusa *and *Rhodochlamys*/*Eumusa *to some 28 Mya (Figure 3, nodes C, D). Within the Clade I, the B genome lineage (*M. balbisiana *species) was the first to diverge, followed by the *M. mannii *lineage, representing the *Rhodochlamys *section, at 20 Mya. Speciation within the A genome lineage (*M. acuminata *species) began 11.4 Mya. The minimum age of *M. ornata*, which appears to belong to the A genome group within *Eumusa *section, is estimated to be 8.7 Mya (Figure 3; node I).

Although *M. mannii *is an "imperfectly understood small species up to 1.3 m high with purplish-red bracts that do not curl back" [75], it undoubtedly belongs to section *Rhodochlamys*, which is confined to the monsoon-affected areas of Southeast Asia. Its characteristic dry-season die-back is presumably an adaptation to drought, and contrasts with the behavior of the *Eumusa *species endemic to the same geographical region, which survive the dry season, although often in poor condition [71]. The monsoon regime was established following the formation of the Himalayas and the Tibetan plateau, and is thought to have stabilized in its current form around 20-25 Mya [76]. The estimated divergence date of *M. mannii *(20 Mya, Table 5) could therefore reflect an adaptation to climate change. The later divergence time of the other *Rhodochlamys *member, *M. ornata*, could be explained by its probable derivation from a hybrid between *M. velutina *(section *Rhodochlamys*) and *M. flaviflora*, belonging to a taxon intermediate between *Rhodochlamys *and *Eumusa *[73].

The speciation of the *Callimusa *species can be dated between 8.8 and 28.7 Mya, while the divergence of the *Australimusa *species occurred ~5 Mya (Figure 3, nodes H, J). The relatively recent emergence of the section *Australimusa *is consistent with its perception as an evolutionarily rather young group [77]. Shepherd [73] determined that the "species" within this section behave genetically as a single species, which he therefore designated *Musa textilis *Née. The current phylogeny (Figure 3) supports this view, implying that *M. textilis *could well be the founding species of the entire section. Numerical taxonomy has placed *M. textilis *equidistant from the four *Musa *sections [78]. In this context it is worth noting that robust and sterile diploid hybrids ('Canton') between *M. textilis *(*x *= 10) and 'Pacol' (a form of *M. balbisiana, x *= 11) are common in The Philippines.

The divergence of *M. coccinea *appears to be rather older than that of the members of the *Australimusa *section (Table 5). Unsuccessful attempts to cross two *Callimusa *species *M. coccinea *and *M. borneensis *led Shepherd [79] to suggest that they differentiated from one another long before the evolution of the *Australimusa *species. The seed morphology of *Callimusa *species is very different from that of any of the other *Musa *sections, being cylindrical, barrel- or top-shaped, and marked externally by a transverse line or groove. When ripe, they develop a large, empty chalazal (perisperm) chamber above the groove [10,77]. Although the molecular data alone indicate the paraphyletic position *Callimusa *to *Australimusa *entries (Figure 3), given the above mentioned morphological aspects and the flexibility of the term "section" by Cheesman [3] we believe that merging the two *Musa *sections with *x *= 10, as proposed by Wong *et al*. [10] and indicated by Li et al. [8], is not tenable.

## Conclusions

The gene sequence-based phylogeny presented here provides a substantial insight into the course of speciation within the Musaceae. The data tend to sustain the close relationship of *Rhodochlamys *and *Eumusa *species, supporting the possibility of merging the two sections into a single one. A greater number of species sampled could generate an improved classification, and could help in clarifying the relationship between the species *Rhodochlamys *and *M. acuminata*, as well as to confirm the generic status of *Musella *and *Ensete*. Based on the largest amount of nucleotide characters for Musaceae obtained to date, this study provides the first estimates of divergence times for individual *Musa *sections and genome groups within the Musaceae. Although limited by the number of species sampled from individual sections and subgroups, we provide a plausible reconstruction of speciation events within the Musaceae, a family which has given rise to one of mankind's major crops.

## Authors' contributions

PC carried out the experimental work, participated in the sequence alignment and phylogenetic analysis, and drafted the manuscript. MV participated in the design and coordination of the study, in the phylogenetic analysis and helped to compile the manuscript. EH participated in the sequence alignment and phylogenetic analysis. JD participated in the design and coordination of the study. EDL helped with the taxonomic expertise. JD and EDL revised manuscript critically for important intellectual content. All authors read and approved the final manuscript.

## Supplementary Material

Additional file 1 **Primer sequences used to amplify fragments of the 19 target genes**. Click here for file

Additional file 2 **Topologies of the phylogenies derived from the sequences of individual gene fragments**. Click here for file

Additional file 3 **Phylogeny based on the Bayesian analysis of dataset A**. Click here for file
