# Supplementary material for: A multi gene sequence-based phylogeny of the Musaceae (banana) family
Source: BMC Evol Biol. 2011 Apr 16;11:103. doi: 10.1186/1471-2148-11-103 (PMC3102628; doi:10.1186/1471-2148-11-103)
Supplement: Additional file 1 — Primer sequences used to amplify fragments of the 19 target genes. [file 1471-2148-11-103-S1.PDF]

Supplementary file 1. Primer sequences used to amplify fragments of the 19 target genes.

Gene	Forward primer 5´- 3´	Reverse primer 5´- 3´
g-1	GCAACTTCCAATCCAAAGGA	GCATCAGAGCTGACATGGAA
g-2	TCTGTGCTTCCACTGATGAAA	TGGACTAGGACTCCCTGACA
g-3	CAGAACCCTCCTCCTCTGTCT	GCTGCTATTGGTGGTCCATT
g-4	ACGTCGTCTTGTCCTCTTGG	GCAGGTGACGCAGTCCTT
g-5	CAAAAACAGGAAGCACACCA	TACCTTGGGCCATTGGAGTA
g-6	GGTCTTGATGAACAAGGGCTA	TGTAGCTTGCTGATTCCTTAGATG
g-7	GAACTTGAGAAATCTTGGAAGCA	CGTTCCATTGCATCATCATC
g-8	GAGGAACGAAACCCCTTCC	ATGCATTTTTGATCACCAGTACC
g-9	CAGGCTCCGTGAACAGGTA	CCCAAGGTCGAGAAACAAGA
g-10	GGTGATGGAACCACCACTGT	TTCCGCATTTTCTTTCTCTGA
g-11	CTGAAACATAGACACAGGTCCA	AGGTTGATGGAGGATCCAAT
g-12	TTCCGTGGGTATGGTAGACG	TGAGCCTGGTCGCAAGTAG
g-13	CCATCATCCAGTTGAAGAAGC	CGTGCAGGGAAACTGAACTT
g-14	TGCAAGGATCTATGGGCAGT	AACAGGAAACTGCCATGACC
g-15	CAACCCAGCGTGCTCTTATT	CAGTGGCAGCTCGTAAATCA
g-16	TCCAAATGCTGTGGTTTCTG	CATCATGATCCACAGCTTTTGT
g-17	TGCACATATGAGTGCTTGGAG	CCATCCATCTGGCCATAGAC
g-18	CTCACGCAATCATGGCAAAT	CATTCAGCAGAACCTCAGGA
g-19	TGCTGCAACTGCTCCTAATG	GCTTCTCTTCCCTTGCAGTG
