# Supplementary material for: A multi gene sequence-based phylogeny of the Musaceae (banana) family
Source: BMC Evol Biol. 2011 Apr 16;11:103. doi: 10.1186/1471-2148-11-103 (PMC3102628; doi:10.1186/1471-2148-11-103)
Supplement: Additional file 2 — Topologies of the phylogenies derived from the sequences of individual gene fragments. [file 1471-2148-11-103-S2.PDF]

Supplementary File 2. Topologies of the phylogenies derived from the sequences of individual gene fragments.

gene	MP [§]	ML	BI
g-1*	((((((1,5),2),(3,4),6),(((11,10),9,8),7)),(12,13)),14)	((((((1,5),2),(3,4),6),(((11,10),9,8),7)),(12,13)),14)	(((((((1,5),2),((3,4),6)),(((11,10),(9,8)),7)),(12,13)),14))
g-1	(((((1,5),2),(3,4),6),(((11,10),9,8),7)),12,13)	(((((1,5),2),(3,4),6),(((11,10),9,8),7)),12,13)	((((((1,5),2),(6,(3,4))),((((11,10),8),9),7)),(12,13)))
	(((((1,5),2),(3,4),6),((((11,10),8),9),7)),12,13)		
g-2	((((1,2,5),((3,4),6)),(((9,11),10,8),7)),13,12)	((((1,2,5),((3,4),6)),(((9,11),10,8),7)),13,12)	(((((1,2),5),((3,4),6)),((((11,9),10),8),7)),(12,13))
g-2*	((((1,2,5),((3,4),6)),((((9,11),10,8),7),(12,13))),14)	(((((1,2,5),((3,4),6)),(13,12)),(((9,11),10,8),7)),14)	((((((2,5),1)),((3,4),6)),(12,13)),(((((11,9),10),8),7))),14)
	(((((1,2,5),((3,4),6)),(12,13)),(((9,11),10,8),7)),14)		
	(((((1,2,5),((((9,11),10,8),7),(12,13))),6),(3,4)),14)		
	((((((1,2,5),((3,4),6)),(12,13)),((9,11),10,8)),7),14)		
	((((((1,2,5),((3,4),6)),(12,13)),7),((9,11),10,8)),14)		
	((((((1,2,5),((3,4),6)),(12,13)),7),(((9,11),10),8)),14)		
g-3	(((((1,2,5),3,4),6),((8,(9,(10,11))),7)),12,13)	(((((1,2,5),3,4),6),((8,(9,(10,11))),7)),12,13)	((((((1,5),2),(3,4)),6),((((10,11),9),8),7)),(12,13))
g-3*	((((((1,2,5),3,4),6),((8,(9,(10,11))),7)),(12,13)),14)	((((((1,2,5),3,4),6),((8,(9,(10,11))),7)),(12,13)),14)	(((((((1,5),2),(3,4)),6),((((10,11),9),8),7)),(12,13)),14)
g-4	((((1,2,5,6),(4,3)),10,11,9,8,7),13,12)	((((1,2,5,6),(4,3)),10,11,9,8,7),13,12)	((((((1,5),6),2),(3,4)),(((8,11),(7,9)),10)),(12,13))
g-5	((((((1,5),6),(3,4)),2),((8,9,10,11),7)),12,13)	(((((1,5),6),2,(4,3)),((8,10,9,11),7)),12,13)	(((((1,5),6),((3,4),2)),((((10,11),8),9),7)),(12,13))
	(((((1,5),6),(2,(3,4))),((8,9,10,11),7)),12,13)		
	((((((1,5),6),2),(3,4)),((8,9,10,11),7)),12,13)		
g-5*	(((((((1,5),6),(3,4)),2),((8,9,10,11),7)),(12,13)),14)	((((((1,5),6),(2,(4,3))),((8,10,9,11),7)),(12,13)),14)	(((((((1,5),6),2),(3,4)),((((10,11),9),8),7)),(12,13)),14)
	((((((1,5),6),(2,(3,4))),((8,9,10,11),7)),(12,13)),14)		
	(((((((1,5),6),2),(3,4)),((8,9,10,11),7)),(12,13)),14)		
	(((((((1,5),6),(3,4)),2),(12,13)),((8,9,10,11),7)),14)		
g-6	((((1,2,5,6),(3,4)),(((11,9,10),8),7)),12,13)	((((1,2,5,6),(3,4)),(((11,9,10),8),7)),12,13)	((((((1,6),5),2),(3,4)),((((10,11),9),8),7)),(12,13))
g-6*	(((((1,2,5,6),(3,4)),(((11,9,10),8),7)),(12,13)),14)	(((((1,2,5,6),(3,4)),(((11,9,10),8),7)),(12,13)),14)	(((((((2,6),1),5),(3,4)),((((10,11),9),8),7)),(12,13)),14)
g-7	((((((1,2),6),5),(3,4)),(((10,9,11),8),7)),12,13)	((((((1,2),6),5),(3,4)),(((10,9,11),8),7)),12,13)	((((((1,2),6),5),(3,4)),((((10,11),9),8),7)),(12,13))
g-8	((((1,2,5,6),(3,4)),(7,(8,(9,10,11)))),12,13)	((((1,2,5,6),(3,4)),(7,(8,(9,10,11)))),12,13)	((((((2,5),1),6),(3,4)),((((11,10),9),8),7)),(12,13))
g--9	(((((((1,5),6),2),(3,4)),7),(8,11,10,9)),12,13)	(((((((1,5),6),2),(3,4)),7),(8,11,10,9)),12,13)	((((((1,5),6),2),(3,4)),7),(((11,10),8),9)),(12,13))
	(((((((1,5),6),2),(3,4)),7),(8,11,10,9)),12,13)		
g-9*	((((((((1,5),2),6),(3,4)),(8,11,10,9)),7),(12,13)),14)	((((((((1,5),6),2),(3,4)),7),(8,11,10,9)),(12,13)),14)	(((((((((1,5),6),2),(3,4)),7),(((10,11),8),9)),(12,13)),14))
	((((((((1,5),6),2),(3,4)),(8,11,10,9)),7),(12,13)),14)		
g-10	((((((1,5),2),6),(3,4)),((8,(11,10),9),7)),12,13)	((((((1,5),2),6),(3,4)),((8,(11,10),9),7)),12,13)	((((((1,5),2),6),(3,4)),((((10,11),9),8),7)),(12,13))
g-11	((((((1,2),5),(3,4)),6),((((10,11),9),8),7)),12,13)	((((((1,2),5),(3,4)),6),((((10,11),9),8),7)),12,13)	(((((((1,2),5),(3,4)),6),((((10,11),9),8),7)),12),13)
g-11*	((((((((1,2),5),(3,4)),6),((((10,11),9),8),7)),12),13),14)	(((((((1,2),5),(3,4)),6),(12,13)),(((10,11),9),8),7),14)	((((((((1,2),5),(3,4)),6),((((10,11),9),8),7)),12),13),14)
g-12	((((((1,2),5),6),(3,4)),((((10,11),9),8),7)),12,13)	((((((1,2),5),6),(3,4)),(((10,11),(9,8)),7)),12,13)	(((((1,2),(5,6)),(3,4)),(12,13)),(((((11,10),9),8),7)))
	(((((1,2),(5,6)),(3,4)),((((10,11),9),8),7)),12,13)		
g-13	(((((1,5),2),((3,4),6)),((((11,10),8),9),7)),12,13)	(((((1,5),2),((3,4),6)),((((11,10),8),9),7)),12,13)	(((((1,5),2),((3,4),6)),((((10,11),8),9),7)),(12,13))
g-14	(((((1,2,5),(3,4)),6),(7,((8,10,11),9))),12,13)	(((((1,2,5),(4,3)),6),(7,((8,11,10),9))),12,13)	((((((((1,2),5),(3,4)),6),((((11,8),10),9),7)),12)),13)
	((((1,2,5),((3,4),6)),(7,((8,10,11),9))),12,13)		
g-15	(((((1,2,5),6),(4,3)),(7,((9,11,10),8))),12,13)	(((((1,2,5),6),(4,3)),(7,((9,11,10),8))),12,13)	((((((1,5),2),6),(3,4)),((((9,10),11),8),7)),(12,13))
g-16	((((((1,5),6),2),(3,4)),((10,11,9,8),7)),13,12)	((((((1,5),6),2),(3,4)),((10,11,9,8),7)),13,12)	(((((((1,5),6),2),(3,4)),(12,13)),(((9,8),(11,10)),7)))
g-17	((((((1,6),5),2),(3,4)),((((11,10),9),8),7)),12,13)	((((1,(5,6)),2,(3,4)),((((11,10),9),8),7)),12,13)	(((((5,6),1),((3,4),2)),((((11,10),9),8),7)),(12,13))

gene	MP [§]	ML	BI
	((((1,5,6),2,(3,4)),((((11,10),9),8),7)),12,13)		
	((((1,(5,6)),2,(3,4)),((((11,10),9),8),7)),12,13)		
	((((1,(5,6)),(2,(3,4))),((((11,10),9),8),7)),12,13)		
**g-18**	((((((1,2),5),6),(3,4)),(((11,9,10),8),7)),12,13)	((((((1,2),5),6),(3,4)),(((11,9,10),8),7)),12,13)	((((((1,2),5),6),(3,4)),((((11,10),9),8),7)),(12,13))
**g-18***	((1,2,((((3,4),((((11,9,10),8),7),(12,13))),6),5)),14)	((1,2,((((3,4),((((11,9,10),8),7),(12,13))),6),5)),14)	((((((((1,14),2),5),6)),(3,4)),((((11,10),9),8),7)),(12,13))
**g-19**	(((((1,2,5),6),(3,4)),(((9,(11,10)),8),7)),12,13)	((((1,2,5),6),(3,4),(((9,(11,10)),8),7)),12,13)	((((((2,5),1),6),(3,4)),((((10,11),9),8),7)),(12,13))
	((((1,2,5),6),(3,4),(((9,(11,10)),8),7)),12,13)		
	(((((1,2,5),6),(((9,(11,10)),8),7)),(3,4)),12,13)		
**g-19***	((((((1,2,5),6),(((9,(11,10)),8),7)),(3,4)),(12,13)),14)	(((((1,2,5),6),(3,4),(((9,(11,10)),8),7)),(12,13)),14)	(((((((1,5),2),6),(3,4)),((((10,11),9),8),7)),(12,13)),14)

* including the outgroup *S. nicolai*

[§] all equaly most parsimonious trees are listed

Translate:

1 *M. acuminata* ssp. burmannica
2 *M. acuminata* ssp. zebrina
3 *M. balbisiana* ‘Tani’
4 *M. balbisiana* ‘PKW’
5 *M. ornata*
6 *M. mannii*
7 *M. coccinea*
8 *M. beccarii*
9 *M. textilis*
10 *M. maclayi*
11 Fe’i ‘Menei’
12 *Musella lasiocarpa*
13 *Ensete ventricosum*
14 *Strelitzia nicolai*
