# Supplementary material for: A multi gene sequence-based phylogeny of the Musaceae (banana) family
Source: BMC Evol Biol. 2011 Apr 16;11:103. doi: 10.1186/1471-2148-11-103 (PMC3102628; doi:10.1186/1471-2148-11-103)

Supplementary File 3. Phylogeny based on a Bayesian analysis of dataset A.

Numbers above branches indicate the posterior probabilities.

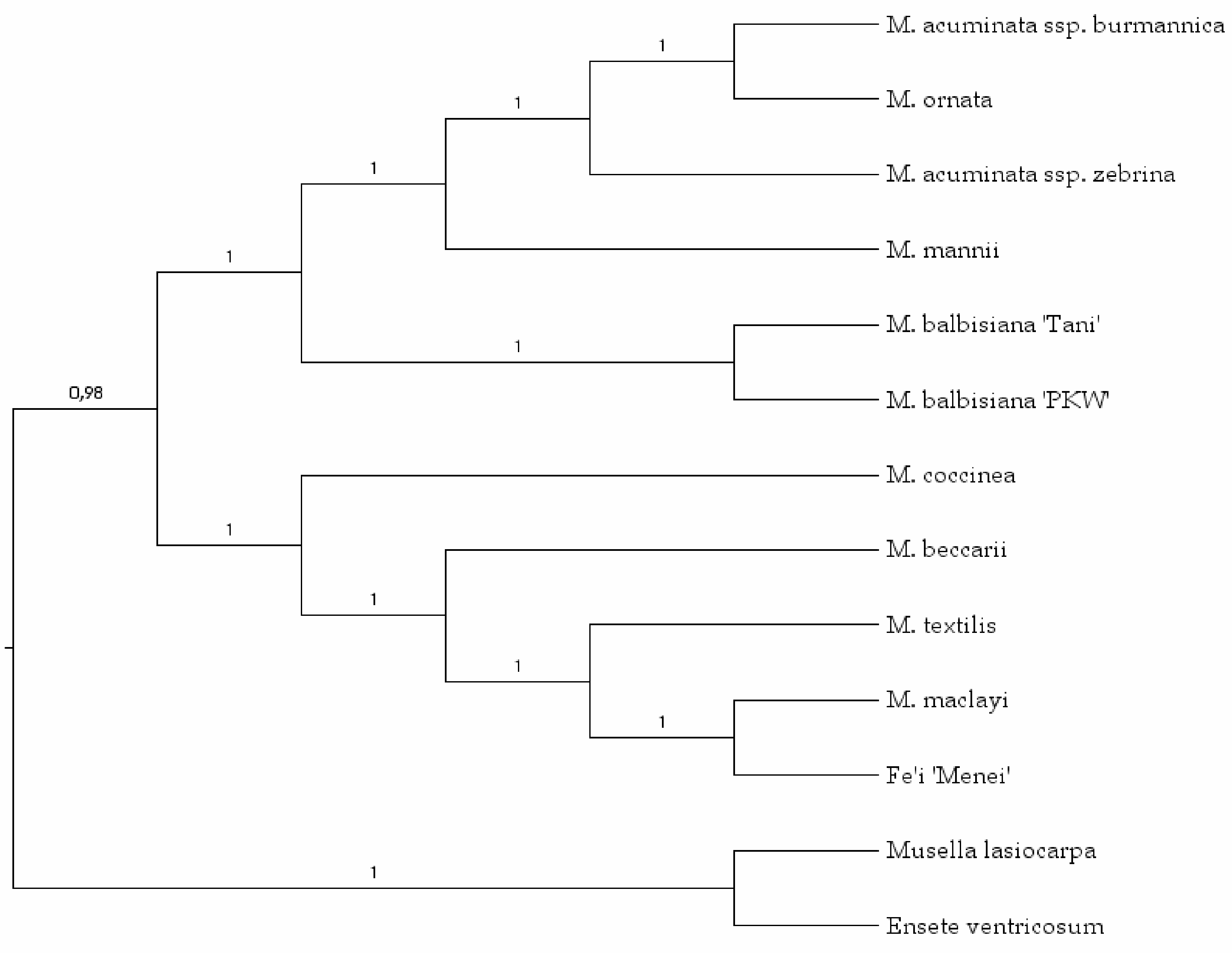

Supplement: Additional file 3 — Phylogeny based on the Bayesian analysis of dataset A. [file 1471-2148-11-103-S3.PDF]
